# Factors that influence an individual’s decision to undergo bariatric surgery: A qualitative systematic review

**DOI:** 10.1371/journal.pone.0334837

**Published:** 2025-10-17

**Authors:** Natasha Keogh, Dawn Horsom, Geraldine Lee, Mohamad M. Saab

**Affiliations:** 1 Catherine McAuley School of Nursing and Midwifery, University College Cork, Cork, Ireland; 2 Bon Secours Hospital, Cork, Ireland; 3 South Tipperary Community Services, Clonmel, Tipperary, Ireland; The University of Edinburgh, UNITED KINGDOM OF GREAT BRITAIN AND NORTHERN IRELAND

## Abstract

**Introduction:**

Obesity is a significant health issue associated with chronic conditions such as cardiovascular disease and type 2 diabetes. Bariatric surgery is the most effective weight loss treatment for obesity. This systematic review aimed to examine factors influencing individuals’ decisions to undergo bariatric surgery.

**Methods:**

This systematic review was guided by the JBI Manual for Evidence Synthesis for Qualitative Reviews and reported using the Preferred Reporting Items for Systematic Reviews and Meta-Analyses checklist. Literature searches were conducted in CINAHL, PubMed, Cochrane Library, APA PsycINFO, and APA PsycArticles. Results were analysed using a meta-aggregative approach. Quality appraisal was conducted using the JBI Checklist for Qualitative Research.

**Results:**

Thirteen studies were included. Health concerns, fears of obesity-related comorbidities, and a desire to improve physical health and quality of life emerged as key motivators to undergoing bariatric surgery. Support from healthcare professionals and family played a crucial role in motivating individuals to consider surgery. Women particularly noted concerns about fertility as a motivator to undergo bariatric surgery. Disclosure of surgery, financial concerns, transport, family and work commitments, and perceived risks of surgery were identified as barriers to undergoing bariatric surgery.

**Conclusion:**

Findings highlight the need for healthcare professionals to adopt empathetic, patient-centred approaches when discussing bariatric surgery. Addressing financial, insurance, and logistical barriers, alongside stigma and family resistance, is essential. Improving patient education, strengthening provider relationships, and offering tailored support can enhance decision-making, access, and long-term outcomes for those considering bariatric surgery.

## Introduction

The World Health Organization [1] defines obesity as a chronic disease characterised by excessive fat that can impair health. Obesity is an increasing health burden. Its prevalence is rising rapidly and is now considered a global epidemic. Adult obesity has doubled worldwide since 1990, while adolescent obesity has quadrupled [1]. In 2022, 2.5 billion adults were overweight; of those, 890 million were living with obesity [1].

Obesity is associated with reduced life expectancy, as well as increased comorbidities and mortality rates [2]. It can impair quality of life, adversely affect vital organs, and contribute to the development of cardiovascular disease, type 2 diabetes, sleep apnoea, osteoarthritis, polycystic ovary syndrome, and hypertension, which in turn may lead to strokes, kidney failure, or death [3]. The effect of obesity on fertility issues in women is well documented. Potential disruptions associated with obesity include irregular menstrual cycles, ovarian dysfunction, endometrial hyperplasia, and malignancy. Obesity can significantly decrease the chances of natural conception, reduce the likelihood of successful fertility treatments, and increase the risks of miscarriage, congenital anomalies, and pregnancy-related complications [4]. It is also associated with sexual dysfunction in women, which tends to improve with weight loss interventions including bariatric surgery [5]. The same can be true for men, with one study reporting that 80% of men presenting with erectile dysfunction were obese [6]. Patients with obesity experience higher healthcare costs and prolonged hospital stay [7]. Obesity can lead to reduced productivity through absenteeism and decreased performance at work [8]. It also increases the risk of unemployment and negatively affects wages [9].

The treatment of obesity-related conditions often requires ongoing medical intervention, including medications, hospitalisation, and surgery. Lifestyle modifications for treating obesity may include physical activity, healthy meal plans, and behavioural therapy [10]. Weight loss diets have a limited effect, with experts estimating that between 80% and 95% of dieters regain the weight they lost [11]. Pharmacological agents such as liraglutide (Saxenda) and Semaglutide (Wegovy) have shown significant effects in obese individuals. These agents have been approved by the National Institute for Health and Care Excellence for use in the United Kingdom’s National Health Service [12,13]. Medical management with GLP-1 receptor agonists such as Ozempic or Saxenda that are used in the management of type 2 diabetes, are clinically proven to reduce obesity via appetite suppression [14]. These drugs stimulate the production of insulin, reduce the production of glucose in the liver, and lower blood sugar levels thus promoting weight loss [15].

Bariatric surgery is a safe and effective intervention for weight loss management when other weight loss methods have been unsuccessful. Like any surgery, bariatric surgery is not without risks; however, these risks are considered low with mortality rates comparable to or lower than those of other elective surgeries [16]. Bariatric surgery can reduce a patient’s risk of premature death by 30% to 50% [17]. Patients often lose 60% of their excess weight within the first six months and 77% by 12 months post-surgery. On average, five years after surgery, patient maintain 50% of their excess weight loss [17]. It is estimated that between 50.5% and 69.2% of excess weight can be lost following bariatric surgery [18]. One of the key findings affecting appetite is that ghrelin production significantly decreases following bariatric surgery [19]. In addition to the reduction in calorie intake post-surgery, changes in taste perception, olfactory sensitivity, food preferences, and aversions may further promote dietary modifications that support weight loss. The main types of bariatric surgery include gastric bypass, sleeve gastrectomy, gastric band insertion, and biliopancreatic diversion with duodenal switch.

The decision to undergo bariatric surgery is often driven by a combination of complex factors such as health concerns, impaired quality of life, and societal pressure. Individuals with obesity are more likely to experience depression and anxiety [20]. Social stigma and discrimination are evident among people who are living with obesity [21]. Understanding decision-making processes and barriers in bariatric care is key for healthcare professionals, as it has the potential to improve patient outcomes. Bariatric surgery, combined with dietary and behavioural modifications, is the clinically effective treatment for individuals with complex obesity. This approach results in significant weight loss and improvements in comorbidities, including diabetes [22]. To the best of our knowledge, no recent reviews have addressed the combined motivators and barriers affecting individuals’ decisions to undergo bariatric surgery. Therefore, the aim of this systematic review was to investigate factors that influence an individual’s decision to undergo bariatric surgery. This review aimed to answer the following questions:

(i) What are the factors that motivate an individual to undergo bariatric surgery?
(ii) What are the factors that prevent an individual from undergoing bariatric surgery?

## Methods

### Design

This systematic review was guided by the JBI Manual for Evidence Synthesis for Qualitative Reviews [23] and is reported using the Preferred Reporting Items for Systematic Reviews and Meta-Analyses (PRISMA) checklist (S1 File) [24].

### Eligibility criteria

The review eligibility criteria were guided by the population, exposure, and outcome (PEO) framework [25]. Inclusion criteria were as follows: Population: Adults (≥18 years of age) of any gender with a body mass index (BMI) of 40 or more or 35 with one comorbidity associated with obesity. This is in line with the International Federation for the Surgery of Obesity guidelines for bariatric surgery [26]. Exposure: Any type of bariatric surgery. Outcomes: Factors that motivate or prevent an individual from undergoing bariatric surgery. As this is a qualitative systematic review, only primary qualitative studies were included to capture the depth and breadth of participants’ experiences. Studies with paediatric patients under the age of 18 years, individuals who do not suffer from obesity, and individuals undergoing other weight loss measures (e.g., pharmacological measures only) were excluded.

### Search strategy

The search strategy was developed in consultation with an academic librarian. First, a scoping search was conducted in Google, Scopus, Cochrane’s CENTRAL database, previous dissertations, the Health Service Executive (Ireland’s public health and social care services provider), the International Federation for the Surgery of Obesity, and Cleveland Clinic to identify relevant keywords. A systematic search was then conducted in CINAHL, PubMed, Cochrane Library, APA PsycINFO, and APA PsycArticles. Synonyms associated with bariatric surgery were searched within the title or abstract fields. Using thesaurus terms for each concept helped identify appropriate subject headings, such as Medical Subject Headings, relevant to each database.

The PEO framework guided the database search, facilitating the effective identification and combination of search terms. Search techniques included the use of phrase searching and truncation (*) to retrieve the most relevant articles. Keywords were searched as follows: (“Bariatric surger*” OR “weight loss surger*” OR “gastric sleeve” OR “gastric bypass” OR “gastric band*”) AND (“Decision making” OR decision-making OR “decision* OR decid* OR choice OR choose OR motivat* OR facilit* OR enable* OR inhibit* OR imped*”). Database limiters were applied for the years 2008–2025. The choice of year limit was guided by a 2007 study conducted by Munoz et al. [27] which, to the best of our knowledge, was the only study that combined motivators and barriers to undergoing bariatric surgery. The search was last conducted on March 22^nd^, 2025. The search strategy can be found in S2 File.

### Study selection

References were imported into Covidence, an online software platform designed to streamline the systematic review process [28]. Duplications were deleted automatically in Covidence. The review aims, questions, and eligibility criteria were shared with a second reviewer. The first and second reviewers independently screened the titles and abstracts of all records. Following the exclusion of irrelevant studies, each potentially eligible study was reviewed in full text independently by both reviewers. Any conflicts identified at either stage of screening were resolved through discussion. A third reviewer was involved if consensus was not reached between the two reviewers.

### Data extraction

Data were extracted into a table adapted from Luzzolino and Kim [29]. The following headings were used: author(s), year, country, aims, design, setting, sample size, strategy, population, data collection, and instruments used. Barriers and motivators were reported within the table. Data extraction tables presented a summary of key study findings. They also offered a structured way to organise and compare data from multiple studies. Data extracted by the first reviewer were cross-checked by a second and third reviewer to ensure consistency and accuracy. The full data extraction table can be found in S3 File.

### Data synthesis

Data from the included studies were synthesised using a meta-aggregative approach, which follows a structured, three-step process [23]. Initially, the two reviewers familiarised themselves with the qualitative findings of each included study, enabling the extraction of relevant findings accompanied by illustrative excerpts. Findings were then grouped into categories through repeated, detailed examination, with each category containing at least two related findings. Subsequently, the categories were synthesised into overarching statements that provided higher-level interpretations. To align the analysis with the review aim and questions, the findings were organised into two overarching themes: motivators for undergoing bariatric surgery and barriers to undergoing bariatric surgery, with sub-themes detailing specific motivators and barriers. A narrative synthesis was then used to present participants’ views, experiences, and opinions under each theme and sub-theme, incorporating illustrative excerpts from the included studies to support and contextualise the synthesised findings.

### Quality appraisal

The JBI Checklist for Qualitative Research was used to appraise the methodological quality of all studies included in this systematic review [23]. This checklist includes 10 questions to assess whether the research methodology is appropriate to the research question, data collection methods, data analysis, interpretation of results, theoretical framework, conclusion, and analysis. Quality appraisal was completed by one reviewer and cross-checked by a second reviewer. Conflicts in quality appraisal were resolved by a third reviewer.

## Results

### Study selection

**Fig 1:**
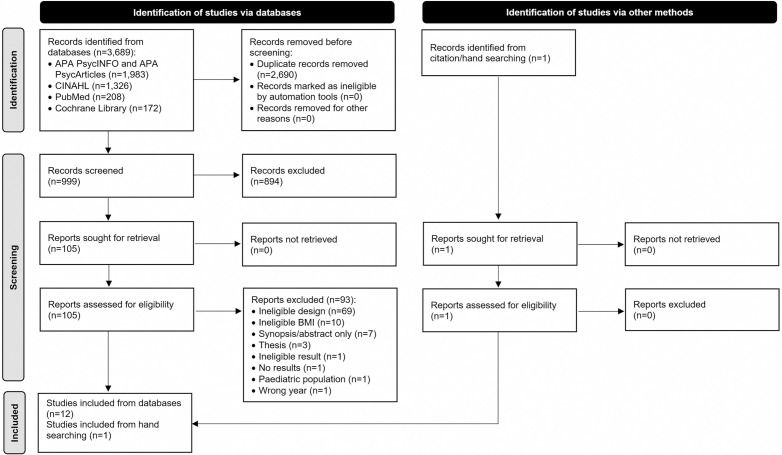
PRISMA flow diagram.

A total of 3,689 records were identified through database searching. Following deduplication, title, abstract, and full text screenings, a total of 12 studies were identified from the database search and one from hand searching the reference lists of the included studies. Therefore, a total of 13 studies were included in this systematic review (Fig 1).

### Study characteristics

Almost half of the studies (n = 6) were conducted in the United States of America (USA). Bariatric centres/clinics were the most represented study settings (n = 3), followed by medical weight loss clinics (n = 2), and surgical centres (n = 2). Sample sizes ranged between 8 [30] and 49 [31] participants with a total of 302 participants. The included studies comprised 202 females and 73 males, with one study not specifying gender for 27 participants. While all studies were qualitative, the exact study design was not specified in seven studies.

Interviews lasted from 10 minutes [32] to 2 hours [33]. Sloan et al. [34] did not report the duration of the interviews. All samples were purposive, except for one study, which used randomly selected participants [30]. Semi-structured interviews were conducted in 11 studies, while focus groups were conducted in two studies [31,35]. Interview guides were used in four studies [36–39]. Only one of the included studies reported having a theoretical underpinning (i.e., self-determination theory) [39]. Characteristics of the included studies can be found in Table 1.

**Table 1:** Characteristics of the included studies (n = 13).

**Country of study**	United states of America (n = 6) Sweden (n = 2) Australia (n = 1) Iran (n = 1) Korea (n = 1) New Zealand (n = 1) The Netherlands (n = 1)
**Study setting**	Bariatric centre/clinic (n = 3) Medical weight loss class (n = 2) Surgical centre (n = 2) Large teaching hospital (n = 1) Obesity clinic (n = 1) Outpatients pre op bariatric (n = 1) Public/Private hospital (n = 1) Research Centre (n = 1) Surgical weight loss centre (n = 1)
**Study design**	Not specified (n = 7) Grounded theory (n = 2) Qualitative descriptive (n = 2) Explorative qualitative (n = 1) Phenomenology (n = 1)
**Theoretical underpinning**	Not specified (n = 12) Self-determination theory (n = 1)
**Total sample size (min-max)**	302 (8–49)
**Gender breakdown**	Female (n = 202) Male (n = 73) Not specified (n = 27)
**Data collection method**	Semi-structured individual interviews (n = 11) Focus groups (n = 2)
**Interview duration (min-max)**	10 minutes–2 hours

### Quality appraisal

All 13 studies had a clear and well-defined research question. Regarding congruity between the stated philosophical perspective and the research methodology, only one study [39] explicitly addressed this criterion. In the remaining studies, this was rated as “not clear,” primarily due to the lack of a clear statement of study design or philosophical/theoretical perspective. Appropriate sampling was used in 12 studies. The sample represented the target population in all studies. In six of the studies, the researchers provided a statement contextualising their cultural or theoretical position within the research [31,32,34,37,38,40]. Three studies failed to address the researchers’ influence on the research [30,35,36]. All participants and their voices were adequately represented in the studies. Ethical considerations and approvals were evident in all but one study [41]. Quality appraisal is presented in S4 File.

### Synthesis of findings

Synthesis of key findings included two themes: (i) Motivators for undergoing bariatric surgery and (ii) barriers to undergoing bariatric surgery. Themes and sub-themes are presented in Table 2.

**Table 2:** Summary of the themes and sub-themes identified from the included studies.

Motivators for undergoing bariatric surgery	Health Activities of daily living Body image Psychological health Advice from healthcare professionals and family members Concerns over family members and oneself Fertility issues among women
Barriers to undergoing bariatric surgery	Disclosure of surgery Terminology around surgery Insurance/ financial concerns Transport Work/ family commitments Fear of death/ unsupported by family due to surgery risk

#### Theme 1: motivators for undergoing bariatric surgery.

##### Health

Health emerged as the primary motivator for undergoing bariatric surgery across all the included studies. Participants spoke of three main concerns: (i) the risk of acquiring a comorbidity due to being obese, (ii) exacerbation of a present condition, and/or (iii) fear of a new health condition. Studies suggested that bariatric surgery had a positive effect on health, particularly concerning cardiovascular health and diabetes [35,42]. Other diseases included diabetes, hypertension, osteoarthritis, and spinal stenosis [33]. Participants in the studies undertaken by Chung et al. [30] and Keledari et al. [41], identified hope for improvement or prevention of comorbidities as main motivators to undergo bariatric surgery. Furthermore, participants in two studies expressed wishes for a healthy life and not to be obese [37,39]. Overall, participants in various studies voiced concerns about avoiding future health problems and wanting to prevent health conditions [30–34,36,38,41,42].

Worsening of present/current conditions was cited where patients spoke of “getting rid of physical health issues such as diabetes or sleep apnoea” (p.731) [36]. Similarly, participants shared their experiences with diabetes: “I’ve type 2 diabetes and I want to get rid of it” (p.106) [31], and noted that patients without any comorbidities often expressed fear of developing them [41]. One participant reported that “health issues” was the most common “tipping point” (p.3) in their decision to undergo surgery [32]. Sharman et al.’s [34] participants talked about chronic diseases such as hypertension and diabetes that kept getting worse and that would be resolved by weight loss surgery. One participant said: “anything I can do to get out of that diabetes loop” (p.3) [34].

One study reported that participants expressed anxiety about losing their lives to obesity, having witnessed such outcomes in their family, and recognised the importance of weight loss [39]. Fear of receiving a new diagnosis of an obesity-related disease and developing a comorbidity was reported in two studies [34,38]. Participants expressed concerns about experiencing the same health issues as their parents. One participant stated: “my mother passed at the age of 54 from a massive heart attack, and she was severely overweight at the time. Yeah, when I felt those real bad pains and I had to go to the hospital and I thought I was having a heart attack, I didn’t want to leave my kids as early as my mother had left us. I’m 43, and my mother passed at 54; there’s 11 years difference” (p.183) [34].

##### Activities of daily living

Participants in 12 of the included studies spoke about the effect of obesity on their lifestyle and daily activities. They hoped bariatric surgery would help them overcome social and physical barriers that they experienced in their daily lives. Many participants reported limitations in engaging in their desired lifestyles or activities [32,33,39,41]. These included challenges with social engagements, eating out, going on vacations, and fitting into aeroplane seats. Participants described the inaccessibility caused by obesity, which prevented them from participating in activities with family and friends, such as going on vacations or dining out. One participant referred to their weight as a social burden stating: “a whole part of your social life is removed if your friends are going to the bar or to dinner and you can’t come” (p.4) [33].

Several studies reported that individuals were unable to participate in activities with their families or peers and often experienced exhaustion or fatigue. These symptoms influenced their decision to undergo bariatric surgery [30,32,36]. One participant stated: “I miss being active, I used to rock climb, scuba dive, I was in the marines. You know, I mean I enjoyed that. Now, I’m exhausted, can’t play with my kids, you know I mean that’s ten and six, that’s the greatest time to be a kid” (p.735) [36]. Participants talked about how activities of daily living such as walking, dressing, cutting their toenails, or picking up items from the floor were problematic because of their weight [31]. They addressed mobility as a motivator: “the weight on my belly was putting too much pressure on my back, and if I dropped something on the floor, it’s known to stay there a week before I can bend down and pick it up” (p.106) [31]. Participants suggested that obesity limited their physical abilities and posed a significant barrier to leading an active life [37,40]. They described putting their lives on hold as a result of becoming obese. They also spoke of how obesity affected them physically and expressed hope that losing weight would restore their true selves, stating: “the real me, is in there somewhere” (p.4) [37].

##### Body image

Most studies (n = 10) addressed body image as a significant motivator for undergoing bariatric surgery. Both, men and women highlighted their desire to improve their body image [31, 33]. Participants reported engaging in body checking and taking extra precautions due to being overweight [35]. As a result, they reported negative feelings and had difficulties accepting their bodies [35,37]. Participants spoke of body image avoidance and challenges regarding travel and eating due to their size [33,35]. One participant expressed their fear of embarrassing their child due to their own obesity: “I was over 40 and I didn’t want to be an embarrassing mother before my child entered school. She’s a daughter. So, I started searching online and on YouTube about dieting because I didn’t want to be an embarrassing mother. That’s when I came across an advertisement on sleeve gastrectomy from a plastic surgery clinic an, and I wondered if such a thing existed. Although I was not a binge eater, I thought, maybe I should just get a consultation (p.47) [30]. Lupher et al. [33] reported that participants expressed a desire to improve their body image, self-image, and self-esteem, with some avoiding having pictures of themselves and experiencing social anxiety due to their weight.

##### Psychological health

Psychological health was identified in nine of the studies as a motivator for undergoing bariatric surgery. Jolles et al. [36] reported that participants highlighted psychological health concerns, including a loss of interest in daily activities due to symptoms of depression, as reasons for undergoing bariatric surgery. Frustration and dissatisfaction were common among participants, with feelings of being fed up due to unsuccessful weight loss attempts or their current weight status emerging as a recurring theme in two studies [31,33]. One participant said: “I was at the end of my tether, tried all diets and they didn’t work” (p.106) [31]. Participants in one study also reported that loss of interest in daily activities or symptoms of depression motivated them to pursue surgery [36]. They viewed obesity as a barrier to leading a normal life and perceived it as carrying a social stigma [36].

Butt et al. [35] found that individuals with obesity engaged in isolating behaviours, which adversely affected their personal relationships and social life. Participants were highly aware of stereotyping associated with obesity, which may contribute to weight-related social stigma. Similarly, participants in another study reported a range of psychological health issues, including obsessive behaviours, paranoid or suicidal thoughts, depression, and a history of self-harm [41]. Feelings of anxiety were reported in the study by Park [39], with health risks serving as a motivating factor for undergoing bariatric surgery. Participants spoke of isolating behaviours: “I didn’t like being out in public because I knew people were looking at me” (p.489) [38]. Relationship difficulties were frequently reported in one study, particularly interactions with the opposite gender [41]. Likewise, another study found that participants faced challenges in relationships, including social isolation and concerns about the impact of weight on sexual performance and the desire for intimate relationships [35].

##### Advice from healthcare professionals and family members

Healthcare professionals such as primary care physicians, fertility specialist consultants, and orthopaedic surgeons played a key role in motivating participants to undergo bariatric surgery in six of the included studies. Chung et al. [30] suggested that healthcare professionals or physicians recommending bariatric surgery significantly influenced patients’ decision to undergo the procedure. Orthopaedic surgeons also recommended bariatric surgery to participants, suggesting that weight loss could negate the need for a knee replacement [33]. Paul et al. [40] discussed how some patients were advised by their healthcare professionals to consider bariatric surgery. One patient said: “so I went to a fertility specialist and gynaecologist with a private practice and she told me to seek gastric bypass surgery because she had seen such good results in her patients” (p. 260) [40].

Family members also played a role in motivating participants to undergo bariatric surgery. Advice from family members was noted in three studies [30,34,39]. Participants in one study discussed the influence of acquaintances who had undergone bariatric surgery: “a friend of mine who used to play sports had lost weight and he told me he had received this metabolic bariatric surgery” (p.48) [30]. Another study also found that family members significantly influenced participants’ decision to undergo bariatric surgery, with three male participants specifically motivated by their wives [31]. The concept of “whanau” which means “family” in Māori was explored by Rahiri et al. [38], with participants highlighting the positive encouragement and support received from their family members: “at times I thought oh stuff it when your numbers are up, it’s up you know? I was going to give up, but my family said, ‘don’t give up’” (p.488) [38].

##### Concerns over family members and oneself

Concerns over family members were expressed in nine studies. Lupher et al. [33] found that several participants pursued bariatric surgery after a family member initiated a conversation about weight and health or suggested bariatric surgery as an option. One participant reported that an older family member expressed concern that he might die before them. Another participant described a conversation where her family gave her surgery as an ultimatum due to her young age and history of losing and regaining over 100 pounds [33]. In the study by Roberson et al. [32], participants viewed bariatric surgery as a last resort, either because previous weight loss methods had failed or their condition had worsened. One participant expressed anxiety about losing their life due to obesity, drawing on experiences with their family [39].

Role modelling motivated individuals to undergo bariatric surgery in five studies. Chung et al. [30] identified participants’ wishes to be a role model for their children: “so I started searching online and on YouTube about dieting because I didn’t want to be an embarrassing mother. That’s when I came across an advertisement on sleeve gastrectomy from a plastic surgery clinic and I wondered if such a thing existed” (p.47). Similarly, one participant in another study referred to role modelling: “I want to set an example for them. It’s not good to be heavy, to take care of yourself, to care about your health” (p.182) [34]. Participants also expressed concern for oneself: “at the rate I was gaining weight I couldn’t see myself; living much longer, cause I was probably going to end up having a heart attack. There wasn’t anything that was going to be worse. I had already given up on the fact that I was going to be dying in the next few months. I thought well okay (surgery) is an option so let’s try” (p.735) [36]. Participants also expressed concerns regarding their personal relationships with themselves, society, and family members [41]. Roberson et al. [32] reported that 10 participants identified family, particularly seeing their grandchildren, as a key motivator for undergoing bariatric surgery. One participant described the incentive as “being there for family” (p.4), while others mentioned wanting to participate in activities such as graduations or babysitting [32].

##### Fertility issues among women

Fertility was a key motivator for undergoing bariatric surgery in three studies. In two studies, participants reported significant issues with their reproductive system [37,40]. Women who were obese or overweight reported experiencing anovulation, infertility, menstrual disorders, difficulties in assisted reproduction, miscarriage, and adverse pregnancy outcomes [31]. Two women reported undergoing surgery to lose weight in order to improve their chances of becoming pregnant. One participant said: “I just wanted a baby so desperately I was prepared to do anything” (p.106) [31]. Participants described obesity as a barrier to starting a family: “…I am dreaming of having children, it’s just not now” (p.4) [37].

In one study, women expressed that their obesity was the primary factor preventing them from safely carrying a foetus to term: “it is not a good combination to be extremely overweight and want a baby” (p.260) [40]. Participants spoke of irregular periods and lack of ovulation: “I weighed a lot, my ovulation did not exist, my menstruation was very irregular and very heavy” (p.260) [40]. Many women who struggled to conceive expressed disappointment at not being able to see a positive pregnancy test: “we tried and tried and tried but never got positive results” (p.260) [40]. Participants also expressed concerns about the potential damage obesity had caused to their bodies, with one stating: “I wasn’t sure if I could produce that hormone again, I was afraid that my body was so destroyed from being overweight” (p.260) [40].

#### Theme 2: barriers to undergoing bariatric surgery.

Only four studies addressed barriers to bariatric surgery. Due to the limited number of studies under this theme, there were insufficient data to develop sub-themes. Consequently, the findings are grouped and presented narratively.

Butt et al. [35] found that finances, transportation, work obligations, and family commitments were barriers to undergoing bariatric surgery. Notably, no illustrative excerpts were provided to support these findings. Similarly, Roberson et al. [32] reported that financial concerns served as a major barrier to undergoing bariatric surgery. Of the 24 participants in the study, nine indicated that a lack of medical insurance prevented them from seeking surgery. Four participants specifically mentioned either “Obamacare” or the Affordable Care Act as being critical to their decision to pursue bariatric surgery. Work obligations also featured as a barrier, with some participants feeling the need to inform their employer about their surgery to account for their absence from work [30]. Disclosure was procedural rather than driven by a desire to share personal health information.

While family support motivated some participants to undergo bariatric surgery, some studies found that family influence also served as a barrier. In the study by Chung et al. [30], participants’ inability to share information with close family members was identified as a barrier in the decision-making process for undergoing bariatric surgery. Participants frequently reported indifference to others’ opinions about their decision, citing a lack of concern for external judgments, a belief that they were not obligated to justify their health choices, and a desire to maintain privacy regarding personal health matters [30]. One participant said: “my family doesn’t know about the surgery. Only my sister and husband know but my son doesn’t know. (I didn’t tell my other two sisters because) There was no need to tell them... Why would I need to tell my son? He’s in his 30s. He was raised by my mother. Thirties is young. But sometimes he is old-fashioned. If I did mention it, he would probably tell me to lose weight through exercise. And it’s difficult for me to talk about it. I didn’t tell them because I didn’t want to listen to them questioning if surgery is needed to actually lose the weight, although I didn’t want to lose the weight” (p. 50) [30]. In keeping with the influence of family, Sloan et al. [34] found that some family members were initially unsupportive of bariatric surgery due to perceived risks. For example, one participant’s son initially opposed her surgery but became supportive five years later, expressing confidence in the technology used to perform the procedure.

The terminology used to describe bariatric surgery was found to be off-putting in certain countries. For example, in Korea sleeve gastrectomy might be interpreted as the removal of one’s stomach. This may be unpleasant and can cause one to dislike this type of surgery [30]. One participant said: “at first the term stomach resection sounded serious. Even for bypass and resection…I felt it was too extreme for me. Do I really need to undergo surgery? Even if the purpose of the bypass is different, when I hear gastric band. I feel offended and imagine my stomach being restricted like you would do with a goose, it’s just the name itself. I hope there is an improvement in the perception of this surgery…” (p.49) [30].

## Discussion

To the best of the authors’ knowledge, this is the first qualitative systematic review of studies that combines an analysis of motivators for, and barriers to undergoing bariatric surgery. Findings indicated that numerous factors motivate the decision to pursue surgery, with health considerations serving as the primary motivator. Indeed, participants across all the reviewed studies identified improving health, gaining more energy for physical activities, and enhancing quality of life as key reasons for undergoing bariatric surgery [43,44].

Obesity is a key driver for life threatening diseases such as cardiovascular disease or certain cancers. The rising prevalence of obesity remains a health concern and should be a top priority in effective treatments in a bid to reduce many of the comorbidities associated with this disease. Bariatric surgery is the most effective method to treat obesity [45].

The current systematic review found that psychological health and advice from healthcare professionals and family members were also key motivators for individuals to undergo bariatric surgery. These findings further reinforce the notion that establishing a strong patient-physician relationship is a key cornerstone in the management of obesity. In Lupher et al.’s [33] study, participants reported trusting their physicians and following their advice if bariatric surgery was recommended as the most appropriate course of action. Patient education is essential in the management of obesity and in discussing available treatment options [46]. Only after being fully informed about the benefits, risks, costs, and alternative medical options can individuals make an informed decision regarding bariatric surgery.

Gender disparity was evident in the decision to undergo bariatric surgery. Over two-thirds of participants in the studies included in this review were female, reflecting the broader trend that approximately 80% of bariatric surgery patients are women and 20% are men [47]. This finding aligns with Farinholt et al. [48], who reported that only 20% of the bariatric surgery population are men. Similarly, Arteburn et al. [49] evaluated 1,368 patients for bariatric surgery and found that men comprised only 18.5% of the study population, highlighting a significant gender disparity among individuals seeking this type of weight loss intervention. These findings are consistent with the current review, in which approximately one-quarter of participants across the included studies were male. Of note, men’s decision to undergo bariatric surgery is often driven by health concerns, such as deteriorating health or physical limitations, whereas women’s motivations may be more influenced by considerations of attractiveness [50].

Barriers to bariatric surgery were explored in only four studies, representing a significant gap in the literature. These included issues relating to disclosure of surgery, terminology used to describe surgery, insurance and financial concerns, transportation, work and family commitments, and fear or lack of family support due to the perceived risks of surgery. Xie et al. [51] highlighted specific insurance-related barriers including insufficient cover or lack of cover for bariatric surgery. Patients often do not fully understand their insurance cover including the extent and the limitations of their cover. In the study by Lim et al. [52], it was found that participants were deterred from undergoing bariatric surgery because of insurance denial and complications. Additionally, out-of-pocket costs for essential services, such as regular dietician appointments, may not be covered by insurance, yet they are crucial for the success of bariatric surgery. Lifelong medications for multivitamin and mineral supplementation to prevent deficiencies post surgeries can be costly. Bariatric surgery costs approximately 17,000–26,000 U.S. Dollars per patient [17]. This financial burden can discourage individuals from undergoing the procedure, particularly those from a lower socio-economic background. Lim et al. [52] suggested that obesity is more prevalent in lower socio-economic areas in Korea. Previously, metabolic and bariatric surgery and related medical care were not covered by the National Health Insurance. However, reimbursement criteria for these procedures were newly established and implemented by the Korean National Health Insurance in 2019 [53]. In Japan, patients with obesity are covered for bariatric surgery; however, gastric bypass is not covered by public health insurance as is often performed in private hospitals [52]. In Australia, bariatric surgery is publicly funded, but the availability of funding is limited. Korda et al. [54] suggested that bariatric surgery in Australia is primarily performed in private hospitals, resulting in substantial out-of-pocket expenses for patients. Unfortunately, those with the greatest need for surgery – primarily individuals with lower incomes who cannot afford private insurance – have the poorest access.

Socioeconomic status and language were not addressed in the studies included in this systematic review. However, these factors were highlighted in the systematic review by Luzzolino and Kim [29]. Additionally, Westerveld et al. [55] identified several environmental and social challenges to bariatric patients. Living in rural areas was found to limit access to healthier foods. Participants from socio-economically disadvantaged areas also faced barriers to physical activity, as fear of crime – such as robbery or assault – deterred them from physical activities like walking.

## Limitations

This systematic review has limitations that should be considered when interpreting its findings.

Although our review was designed to explore both motivators and barriers to undergoing bariatric surgery – as reflected in our aim, review questions, eligibility criteria, and search strategy – only four of the included studies examined barriers. This highlights a gap in the literature. Consequently, our findings may not fully capture the range of factors influencing decisions about bariatric surgery, and the generalisability of our conclusions should be interpreted with caution. Moreover, most included studies were not underpinning by a behaviour change framework, theory, or model, with only one study using self-determination theory [39]. This limits the theoretical contextualisation of motivators and barriers to bariatric surgery. Future qualitative studies should incorporate a behaviour change framework, theory, or model to better interpret such motivators and barriers.

The small sample sizes in the 13 included studies may affect result transferability [56]. Some studies used focus groups, which can introduce bias due to dominant voices influencing others’ responses. In one study, participants declined audio recording, which may have affected data accuracy [41]. Over half of the included studies (n = 7) did not specify the exact qualitative design, and only one study reported a philosophical perspective [39], resulting in “not clear” judgments for the first item of the JBI Checklist for Qualitative Research. This limits the ability to fully assess methodological rigour and may influence the reliability and interpretation of the review findings.

Database limitations and inclusion of only English-language studies might have led to omitting potentially eligible studies. Inconsistent reporting of socio-demographic factors across studies limited the understanding of their influence on bariatric surgery decisions. Finally, quality appraisal was conducted by a single reviewer and cross-checked by a second, rather than independently assessed by two reviewers, which is the gold-standard approach. This minor limitation may introduce a risk of bias.

## Conclusion

This systematic review reveals key factors influencing decisions to undergo bariatric surgery. Health concerns emerged as the primary motivator, with participants seeking to address comorbidities, mitigate health risks, and enhance their quality of life. Body image, psychological well-being, and family considerations also played a significant role in motivating individuals. This systematic review highlights how patients’ insights surrounding obesity can be used to guide actions and to encourage clinicians to provide empathic care to patients with this disease. Barriers to accessing bariatric surgery included financial constraints, lack of insurance coverage, work-related obligations, and logistical challenges. Cultural perceptions, stigmatising language, and resistance from family members also presented obstacles. Findings from this review underscore the importance of a comprehensive, empathetic approach by healthcare professionals when addressing motivators and barriers. Providing tailored support, using inclusive language, and improving access to resources may facilitate informed decision-making and potentially lead to better outcomes for patients considering bariatric surgery.

## Supporting information

S1 File PRISMA 2020 checklist. (DOCX)

S2 File Search strategy. (DOCX)

S3 File Data extraction table. (DOCX)

S4 File Quality appraisal. (DOCX)
