# Supplementary material for: Factors that influence an individual’s decision to undergo bariatric surgery: A qualitative systematic review
Source: PLoS One. 2025 Oct 17;20(10):e0334837. doi: 10.1371/journal.pone.0334837 (PMC12533836; doi:10.1371/journal.pone.0334837)
Supplement: S2 File — (DOCX) [file pone.0334837.s002.docx]

**Supporting Information File 2**. Search strategy

| **Database** | **Search terms** |
| --- | --- |
| CINAHL | (“Bariatric surger*” OR “weight loss surger*” OR “gastric sleeve” OR “gastric bypass” OR “gastric band*”) ) OR AB ( (“Bariatric surger*” OR “weight loss surger*” OR “gastric sleeve” OR “gastric bypass” OR “gastric band*”) )  AND  "decision making" OR decision-making OR decision* OR choice OR choose OR motivat* OR facilit*OR enable*OR inhibit *OR imped* OR perceiv*OR percept* Or attitude*OR belie*OR view* OR opinion* ) OR AB ( "decision making" OR decision-making OR decision* OR choice OR choose OR motivat* OR facilit*OR enable*OR inhibit *OR imped* OR perceiv*OR percept* Or attitude*OR belie*OR view* OR opinion* |
| PubMed | Decision making  AND  Bariatric surgery |
| Cochrane Library | Decision making  AND  Bariatric surgery |
| APA PsycArticles | “Decision making” OR decision-making OR decision* OR decid* OR choice OR choose OR motivat* OR facilit* OR enable* OR inhibit* OR imped*). ) OR AB ( (“Decision making” OR decision-making OR decision* OR decid* OR choice OR choose OR motivat* OR facilit* OR enable* OR inhibit* OR imped*) )  AND  “Bariatric surger*” OR “weight loss surger*” OR “gastric sleeve” OR “gastric bypass” OR “gastric band*”) ) OR AB ( (“Bariatric surger*” OR “weight loss surger*” OR “gastric sleeve” OR “gastric bypass” OR “gastric band*” |
| APA PsycInfo | “Decision making” OR decision-making OR decision* OR decid* OR choice OR choose OR motivat* OR facilit* OR enable* OR inhibit* OR imped*). ) OR AB ( (“Decision making” OR decision-making OR decision* OR decid* OR choice OR choose OR motivat* OR facilit* OR enable* OR inhibit* OR imped*) )  AND  “Bariatric surger*” OR “weight loss surger*” OR “gastric sleeve” OR “gastric bypass” OR “gastric band*”) ) OR AB ( (“Bariatric surger*” OR “weight loss surger*” OR “gastric sleeve” OR “gastric bypass” OR “gastric band*” |
