# Supplementary material for: Factors that influence an individual’s decision to undergo bariatric surgery: A qualitative systematic review
Source: PLoS One. 2025 Oct 17;20(10):e0334837. doi: 10.1371/journal.pone.0334837 (PMC12533836; doi:10.1371/journal.pone.0334837)
Supplement: S3 File — (DOCX) [file pone.0334837.s003.docx]

**Supporting Information File 3.** Data extraction table (N=13)

| **a) Author**  **(year)**  **b) Country** | **Aim** | **a) Design**  **b) Setting**  **c) Theoretical underpinning** | **a) Sample size**  **b) Sampling strategy** | **a) Age**  **b) Gender**  **c) Ethnicity**  **d)Average BMI kg/m^2^** | **a) Data collection**  **b) Instruments** | **Findings relating to barriers.**  **1. Disclosure of surgery**  **2. Insurance, financial concerns 3. Transport**  **4. Work, family commitments**  **5. Fear of death, lack of support** | **Findings relating to motivators.**  **1. Health**  **2. Daily Living**  **3. Body image**  **4. Psychological Health**  **5. Advice family and health professional**  **6. Concerns family/oneself**  **7. Fertility** |
| --- | --- | --- | --- | --- | --- | --- | --- |
| a) Butt et al. (2020)  b) USA | To evaluate the phenomena body image in patients before & after bariatric surgery | a) Phenomenology  b) Surgical Weight  Loss Clinic  c) NR | a) n= 13  1 excluded as revision surgery was initially 14  b) purposeful | a)18 years +  Mean age 47.57  b) female  (n=11)  male  (n=2)  c) Hispanic  Caucasian  Black  African  d) BMI=46.36 | a) Focus Groups conducted 60 minutes interviews based on surgical status pre and post-operative perception of body image. Each focus group was digitally audio recorded and transcribed verbatim. Collected Jan to Dec 2019  b) Semi-structured  with prompting  questions to encourage discussion among participants | 1. NR  2-4. Finance, Family commitments,  transportation and  work obligations mentioned as barriers to surgery.  5. NR | 1. Heavily endorsed health concerns as a driving factor to undergo bariatric surgery. (p.3)  2. Physical concerns (p3) and participating in activities with family/peers.  3. Body avoidance is presented with challenges in relation to travel and eating due to their size. Body checking, body avoidance extra consideration due to weight or body image. (p.3)  4. Psychological Aspects- social behaviours including isolating behaviours and impact of weight on sexual performance. Isolating behaviours & negative impact on relationships. Negative cognition and affect. Difficulty accepting their body image mentally. Patients reported denial & disbelief at their current weight suggesting disconnection between oneself and his/her shape or size.” Mind reading “, reflects ores personal fear regarding perceptions of others. Patients reflected stereotyping regarding being overweight the patients voiced these ideas/beliefs fuels social stigma, negative feelings, and notions of acceptance. Desire to improve self -perceptions (p.3).  5-7. NR |
| a) Chung et al. (2023)  b) Korea | To further understand patients’ potential barriers who received metabolic bariatric surgery (MBS) and increase accessibility of MBS | a) Grounded theory  b) Out -Patients clinic participants who received metabolic weight loss surgery  c) NR | a) n=8  b) Selected randomly | a) 26-56 years  b) Female  (n=5)  male  (n=3)  c)NR  d) 50 | a) In -Depth Interviews conducted in July for a duration of 1 month. Questions were based on life before surgery, and life after surgery lasting 1-2 hours done on 1:1 and in-person. Separated in 2 groups sleeve and bypass group. Data collected mid July 2022 X 1 month.  b) Semi Structured Questions regarding life before and decisions process. Mind mapping used and each participant was requested to summarize their interview using this technique. This method helps verifies the interviewees expressed their feelings during the interview. | 1. Disclosure of undergoing bariatric surgery.  Terminology of surgery was off putting in Korea, “sleeve gastrectomy” might be interpreted as “removing the stomach” (P.54*)*  2-5. NR | 1. Worsening diabetes/hypertension (p.46). Improvement of chronic illness diabetes  2. Experience of sudden fatigue (p.48)  3. Dissatisfaction of body image/embarrassment  4. NR  5. Healthcare personnel= primary care physician, “the doctor words were it’s the best” (p.48) patients commented my doctor from my neighbourhood recommended the surgery (p.48) Mass media tv, social 2 networks. “One day while watching YouTube I became across a video saying getting gastric bypass could improve my diabetes” (p.47)  6. Failure at self-control. Role model for one’s children. “didn’t want be an embarrassing mother” Children feeling “to their parents being obese” (p.47) Acquaintances i.e. friend/family successful with bariatric surgery, “a friend of mine had lost weight and he told me he had received weight loss surgery” (p.48). Family/in-laws, “mother-in law would say hurtful things like her son married a fat pig” (p.47)  7. NR |
| a) Jolles et al. (2019)  b) USA | To identify motivators of males who undergo bariatric surgery. | a) Not specified  b) Veterans from two Medical Centres who attended medical weight loss management classes on exercise, nutrition and goal setting.  c) NR | a) n=25  n= 14 for bariatric surgery.  n= 11 weight management program.  b) Purposeful sample. | a) Mean age  58.7  b) Males  only  c) Black 14%  White 79% Other % in weight management program.  d)BMI ≥40 or 35-39.9 1 co-morbidity | a) semi-structured interviews lasting 60 minutes with male veterans. Collected data was from January 2011 to June 2016. Interview guides were used Participants were asked to describe their experience with weight loss programs and weight loss treatment options and their motivation for pursuing bariatric surgery  b) Phone call or  in -person interviews. Verbal consent was obtained for phone consult and written consent for in-person interviews. | NR | 1. Improving physical health. Patients spoke of “getting a better handle” (p.731) or “controlling their diabetes”. Patients expressed “getting rid of” (p.731) of physical health issues such as diabetes and sleep apnoea.  2. Enhancing quality of life. Fear of death “feelings of going to die if did not lose weight” (p.732). Motivated by physical health and independence (p.733).” I miss being active, I used to rock climb and scuba dive and now I’m exhausted” (p.735)  3. Desire to improve body image, Being comfortable in my own skin”. (p.735)  4. Enhance psychological health. Patients voiced their loss of interest in undertaking daily activities or symptoms of depression to pursue surgery. Desire to improve their body image and self-esteem was mentioned. Patients saw obesity as a “barrier” (p.731) to one’s self-esteem and leading a normal life. Obesity as a “stigma “was a factor in patients who were interested in bariatric surgery. “I couldn’t see myself living much longer” (p.735) So I thought surgery is an option so let’s try it” (p.735)  5-7. NR |
| a) Keleidari et al. (2016)  b) Iran | To explore the aspects of quality of life in people with obesity who decide to have bariatric surgery | a) Not specified  b) Obesity Clinic  c) NR | a) n=38  b) Purposeful sample | a) Mean age  35.29.  b) Women  n=27  Men  n=11.  c)NR  d)42.83 | a) Face to face interviews. Time not documented. Length of collection from 2014-2015.Interviews took place the day before their surgery.  b) open ended questions and directional content analysis of data was completed. Some opened-ended questions included “Why did you decide to treat your obesity?” and “what are your expectations of surgery?”. | NR | 1. Physical Health “pain and fatigue” or “Fear of getting comorbidity” (p.2).  2. Physical daily tasks Environment “cloth style and transport” most often mentioned (p.2).  3. Environment body image “cloth Style (p.2).  4. Psychological health “self-esteem”, feelings, and “medication” (P2).  5. NR  6. Social relationships problems, “personal relations “including self, society and family members” (P.2) “relation with the opposite gender” (P.2)  7. NR |
| a) Leclercq et al. (2021)  b) The Netherlands | To gain an understanding of the perceptions and experiences of young adults undergoing bariatric surgery | a) Not specified  b) Large teaching hospital with a bariatric department certified as European Centre of Excellence  c) NR | a) n=27  fully completed the study  b) Purposeful sample | a)18-25 years. Mean age 23.1  b) NR “Mostly female”.  c)NR  d)Mean BMI=43.3 | a) By telephone/interview  in person in the hospital setting no times lengths available. All interviews were performed individually to allow to gather detailed information.3 main aspects were discussed education in the informed consent domain pre-operative expectations and experiences and personal (un) certainties undergoing bariatric surgery. Data collected from October 2017 to April 2018.  b) An oral survey that consisted of closed-ended questions as well as open-ended in the form of a semi-structured interview. | NR | 1. Positive effects on Type 2 diabetes. (p.44)  Positive effects on cardiovascular risk (p.44)  Article main topics education/perioperative expectation/experiences and (un)certainties undergo bariatric surgery. |
| a) Lupher et al. (2022)  b) USA | To determine what factors, influence an individual’s decision to undergo bariatric surgery | a) Grounded theory analysis approach  b) Accredited bariatric centre in Michigan  c) NR | a) n=30    b) Purposeful sample pre bariatric surgery class | a)18 years +  b) Female  (n=24).  Male  (n= 6).  c) White  (n= 24)  Black  (n=6)  d)Mean BMI =46.8-48.1 | a) semi-structured interviews lasting 50 minutes to 2 hours. Recruited from 2013-2014). Participants were recruited from a provider referral. Participants were required to be scheduled for bariatric surgery and no history of bariatric surgery previously.  b) open-ended questions using a conversation format to obtain details and description. Pre-surgery interviews took place at the surgical centres. Interview topics covered diet behaviours, weight management and loss and surgery experiences. Participant were asked to reflect on their decision to undergo bariatric surgery in the 1^st^ interview and their choice of surgical procedure I the 3^rd^ interview, all interviews were audio recorded. | NR | 1. Patients reported “health concerns were leading motivator”, (p.4)  Avoidance of future health problems “wanted to avoid health conditions observed by family members” (p.4).  2. Lifestyle and interference with activities. Almost all participants discussed limitations in desired lifestyles/activities. Social activities, inaccessibility to dining or vacations /fitting in airplane seat. Referring to obesity as “a weight burden” and deterrent from leaving the house” (p.5)  3. Body image focusing on “wanting to be skinner” (p.5)  4. Frustration with current weight Unsuccessful diets/weight loss history,” Fed up” or had enough”, I just can’t do this anymore”. (p.5).  5. Health care providers suggestions to have surgery or orthopaedic surgeon suggest weight loss surgery would eliminate a knee replacement (p.5)  6. Family & friends support regarding surgery decision making process. Family concern worried one will die before them/ gave surgery as a “ultimatum” (p.5) Role Modelling, participant expressed their wish to be “Role model “(p.5) “wanting to achieve a healthy weight and positive dietary behaviours to influence their children” (P.5)  7. NR |
| a) Nilson-Condori et al. (2019)  b) Sweden | To explore the motives behind young women, wish to have bariatric surgery and their expectations on future fertility. | a) Not Specified  b) Single centre in Sweden for patients who were undergoing bariatric surgery in 1-3 weeks times  c) NR | a) n=12  b) Purposive  sampling | a) 20-35 years. Mean age 27  b) All female  c)NR  d)41.6 | a) Semi-structured interviews completed. Interviews were completed either in a hospital setting or in the comfort of the participants’ home if desired. Topics included decision-making to have bariatric surgery, psychological aspects focusing on reproduction and fertility and expectation on surgery and future fertility and surgery expectations.  b) semi-structured using an interview guide. The length of interviews was ranging from 38 -95 minutes and the average lasting 54 minutes. Collection of data was April 2016 to March 2017.Interviews were completed 103 prior to surgery | NR | 1. Health- patients wished to be “a better me” (p.3).  2. Daily activity patients spoke of “how obesity inhibited their life physically” (p.3).  3. Self-image Patients reported they didn’t recognise the “other “person that had been overweight.  4. Obesity negatively affects self-esteem. Depression and anxiety were reported (p. 4).  5. NR  6. Obesity as an obstacle in moving forward to have a family (p.4).  7. “The thought of having a family also meant a dream of being a healthy parent” (p.4) |
| a) Park (2015)  b) USA | To examine how obese individuals, get their motivation to undergo bariatric surgery | a) Not specified  b) Weight loss classes in the USA  c) Self-determination theory | a) n=14  b) Purposeful Sampling | a) 31-61 years  b) Female  (n=11)  Male  (n=3)  c) NR  d) Mean BMI  50.2 | a) Semi-structured, in-depth were completed at the participants location which was convenient to them. Interview took 1 hour approximately on average /mean time, however interview ranging from 45 minutes to 2 hours. No data time collection specified  b) Ten open ended questions were included in the interview schedule. If additional questions arose the researcher would follow up with the interviewee and clarify what was necessary. | NR | 1. Health concerns -anxiety about losing their life for being obese (p.803).  2. Participants felt it was important due to environmental demands.  3. Body image- “I have a boyfriend; I want to like the way I look in clothes (p.823).  4. Anxiety for health risks acted as a driver (p.803)  5.Healthcare professional comments such as “her nephrologist was very blunt and straightforward and to the point with her that was for me having to deal with the reality of dying” (p.803).  Acquaintances “2 of my sisters and niece have actually had a gastric bypass” (p.803).  6. Family reasons “to watch her son grow up” (p.803)  7. NR |
| a) Paul et al.  (2022)  b) Sweden | To explore the expectations and experiences of women who wish to have children as a motivation to undergo bariatric surgery | a) Explorative qualitative  research  b) Single surgical centre in Sweden in a preoperative health survey who had the desire become pregnant as their reason to undergo bariatric surgery  c) NR | a) n=14  b) Purposeful | a)18-45)  b) Female  only  (n=14)  c) NR  d) Mean 41.8 | a) interviews by  telephone/digital zoom meeting x 1 (covid 19 Pandemic). Interview minutes were between 11- 43 minutes with the  mean interview time is 23 minutes. All interviews were carried out individually and, in the participants, home as it was during the Covid 19 pandemic  b) Using an interview guide and open -ended questions. Main topic included experiences concerning the desire to have children and infertility issues, the surgical impact on the possibility of having children, health care contacts in connection with these difficulties and post operative effects on life. | NR | 1. Health concerns, “Everything is your fault if you overweight”. (p.259)  2. Physical daily Activities Obesity as a hindrance to lead an active life (p.258)  3. Body image- Lack of femininity, “It is not a good idea to be overweight and want a baby” (p.259)  4. Psychological health Everything is your fault if you overweight”. Obesity is blamed on whatever problem one experience even if not related. (p.259)  5. Health professionals advise” So I went to a fertility specialist and gynaecology and told me to seek bariatric surgery” (p.258)  6. NR  7. Irregular periods/lack of ovulation. “My ovulation did not exist “(p.259).  Motherhood. “We tried and tried and tried but never got positive results” (p.258) |
| a) Rahiri et al. (2019)  b) New Zealand | Aim to explore motivation of Māori women in coming forward for bariatric surgery | a) Not Specified  b) Māori Research Centre  c) NR | a) n=24  b) Purposeful sampling | a) Mean =45.3  b) Female  (n=22)  Male  (n=2)  c)Caucasian (n=11)  Black  (n=12)  Latino  (n=1)  Multiracial (n=1)  d) BMI+ 51.7 | a) Semi-structured Interview.no times given. Participants were Māori women who had primary bariatric procedure performed from January 2010-to 2014 in the comfortable of their own home.  b) Interviews were conducted using an interview guide, this guide was not pilot tested. | NR | 1. new diagnosis of an obesity related disease. Acquiring a Co - morbidity alarm bells. “They (GP) were saying I was going to be pre-diabetic” (p.487)  2. A better quality of life. Addressing life-long habits that contributed to weight gain were noted.  3. A lifetime of “fattism”.” Being fat “(p.488). Size, shape and contour. Shame. Social isolation. Wearing winter clothes in summer. Discrimination.  4. “I didn’t like being out in public because I knew people were looking at me (p.489).  5. “The orthopaedic surgeon said we can see your trying -here an option” (P489).  6.Whanau =meaning family. “I was going to give up, but my family said don’t give up” (p.488)  7. NR |
| a) Robersons et al. (2016)  b) USA | To identify factors that “tipped the scales”, in the patients experience to move ahead with bariatric surgery | a) Qualitative descriptive  b) Bariatric clinic  c) NR | a) n=24  b) Purposeful sampling | a) Mean = 45.3  b) Female (n=22)  Male  (n=2)  c)Caucasian  n=11  Black n= 12  Latino n=1  Multi-racial  n=1  d) BMI+ 51.7 | a) Semi-structured Interviews. Time 10-45 minutes. Patients were at their decision visit to establish their decision to move forward with surgery, Data was collected from December 2014 to January 2015  b) Questions /probing. The main question was “Tell me what made you decide to have bariatric surgery” (P.2). Other probing questions were based on factors that were identified by the patient | 1. NR  2. financial concerns/Lack of medical insurance 9/24 no insurance prohibits them seeking surgery (P4)  3-5. NR | 1. Health Issues-Patient spoke about chronic conditions such diabetes/hypertension (p.3).  2. Low energy levels- interfered with work, social and physical activity” (p.3).  3-5. NR  6. Role modelling-setting an example for children. “Being there for family”. Support from family and friends. Seeing one’s grandchildren served as a strong motivator to live longer (p.488)  7. NR |
| a) Sharman et al. (2016)  b) Australia | To examine Australian patients’ motivations for bariatric surgery | a) Not specified  b) 2 public hospitals and 1 private hospital  c) NR | a) n=49  b) Stratified random approach sampling strategy | a) Mean age 55.  b) Female  (n=32)  Men  (n=17)  c) NR  d) BMI 46 | a) Semi- Focus groups 1.5 hours. Study was conducted between August and October 2014.  b) Categories groups same sexes and surgery funding type or whether participants were short listed for or had undergone surgery to explore differences in this data. Separated into 2 categories groups same sexes and surgery funding type or whether participants were short listed for or had undergone surgery to explore differences in motivation for surgery. | NR | 1. Health- “I have diabetes type 2” (p.106).  2. Mobility the “the weight on my belly was putting too much pressure on my back” (p.106)  3. Body image-both men and women talked about perceptions of their physical appearances (p.106).  4. Psychological – “I couldn’t continue the way I was, because of the way I felt about myself and the way other people felt about me” (p.106).  5.Health professionals. People stated it was via their GP that they 1st learnt of bariatric surgery and were encouraged by their GP to undergo surgery. (p.106)  Family and close friends’ previous successful bariatric surgery. “Experiences of being the best thing in their lives (p.106)  6.Family- discussion on the significant others were discussed .3 men said their wives had encouraged them to have surgery. (p.106) “Wanting to see their grandson grow up” (p.106)  7. Fertility 2 women spoke of having surgery to lose weight to improve their chances of getting pregnant (p.106) |
| a) Sloan et al. (2020)  b) USA | Aim to explore the family’s influence on patients’ decision to undergo bariatric surgery | a) Qualitative descriptive study  b) A large bariatric clinic  c) NR | a) n =24  b) Purposeful sampling | a) mean=45 years  b) female (n=22)  male (n=2)  c)NR  d)BMI mean 51.7 | a) Interview, no time identified. Examined the primary transcripts to determine the influence family had on decisions to undergo bariatric surgery  b) secondary analysis of collected data from interviews. Limited questions asked. Interview mainly focused on what were the motivators for undergoing bariatric surgery. | 1-4. NR  5.Unsupported family members due to Risk of surgery | 1. Health concerns having the same health issues as their parent, my mother passes away from a massive heart attack, she was severely overweight” (p.185)  2. Participants unable to participate in social aspects of daily lives (P184)  3-5. NR  6. Role Modelling- “I want to set an example for them it’s not good to be heavy” (p. 183)  Family quality time with one’s children “the biggest thing that ever me want to lose weight is when my baby boy was running in the street, and I could hardly catch up with him” (p.183) family support for procedure was also mentioned. Martial and family factors include “he really nagged me”  (p.184) _to consider bariatric surgery (p.184)  7. NR |

BMI=Body Mass Index, NR =Not Reported, USA=United States of America
