# Supplementary material for: Factors that influence an individual’s decision to undergo bariatric surgery: A qualitative systematic review
Source: PLoS One. 2025 Oct 17;20(10):e0334837. doi: 10.1371/journal.pone.0334837 (PMC12533836; doi:10.1371/journal.pone.0334837)
Supplement: S4 File — (DOCX) [file pone.0334837.s004.docx]

**Supporting Information File 4.** Quality appraisal using the JBI Checklist for Qualitative Research

| **Items** | Butt et al. (2020) | Chung et al. (2023) | Jolles et al. (2019) | Keleidari et al. (2016) | Leclercq et al. (2021) | Lupher et al. (2022) | Nilsson et al. (2019) | Park (2016) | Paul et al. (2023) | Rahiri et al. (2019) | Roberson et al. (2016) | Sharman et al. (2016) | Sloan et al. (2020) |  |
| --- | --- | --- | --- | --- | --- | --- | --- | --- | --- | --- | --- | --- | --- | --- |
| Is there congruity between the stated philosophical perspective and the research methodology? | NC | NC | NC | NC | NC | NC | NC | ✓ | NC | NC | NC | NC | NC |  |
| Is there congruity between the research methodology and the research question or objectives? | ✓ | ✓ | ✓ | ✓ | ✓ | ✓ | ✓ | ✓ | ✓ | ✓ | ✓ | ✓ | ✓ |  |
| Is there congruity between the research methodology and the methods used to collect data? | ✓ | ✓ | ✓ | ✓ | ✓ | ✓ | ✓ | ✓ | ✓ | ✓ | ✓ | ✓ | ✓ |  |
| Is there congruity between the research methodology and the representation and analysis of data | ✓ | ✓ | ✓ | ✓ | ✓ | ✓ | ✓ | ✓ | ✓ | ✓ | ✓ | ✓ | ✓ |  |
| Is there congruity between the research methodology and the interpretation of results? | ✓ | ✓ | ✓ | ✓ | ✓ | ✓ | ✓ | ✓ | ✓ | ✓ | ✓ | ✓ | ✓ |  |
| Is there a statement locating the researcher culturally or theoretically? | NC | X | X | X | X | X | ✓ | X | ✓ | ✓ | ✓ | ✓ | ✓ |  |
| Is the influence of the researcher on the research, and vice- versa, addressed | X | NC | NC | ✓ | ✓ | ✓ | ✓ | ✓ | ✓ | ✓ | ✓ | ✓ | ✓ |  |
| Are participants, and their voices, adequately represented? | ✓ | ✓ | ✓ | ✓ | ✓ | ✓ | ✓ | ✓ | ✓ | ✓ | ✓ | ✓ | ✓ |  |
| Is the research ethical according to current criteria or, for recent studies, and is there evidence of ethical approval by an appropriate body? | ✓ | ✓ | ✓ | NC | ✓ | ✓ | ✓ | ✓ | ✓ | ✓ | ✓ | ✓ | ✓ |  |
| Do the conclusions drawn in the research report  flow from the analysis, or interpretation, of the data? | ✓ | ✓ | ✓ | ✓ | ✓ | ✓ | ✓ | ✓ | ✓ | ✓ | ✓ | ✓ | ✓ |  |
| CODES FOR DIAGRAM: ✓ = YES, X = NO, NC =NOT CLEAR | | | | | | | | | | | | | | |
